# Physical Training Is a Potential Modifier of Risk for Contrast-Induced Acute Kidney Injury in Diabetes Mellitus

**DOI:** 10.1155/2020/1830934

**Published:** 2020-11-17

**Authors:** Sheila M. F. Couto, Douglas I. Machado, Carolina Conde, Vinicius C. Silva, Adriana A. Souza, Karina B. Peres, Beatriz A. Brandi, Maria De Fátima F. Vattimo

**Affiliations:** Experimental Laboratory of Animal Models, School of Nursing, University of Sao Paulo, Sao Paulo 05403-000, Brazil

## Abstract

**Background:**

Iodinated contrast (IC) is a leading cause of hospital-based acute kidney injury (AKI). Contrast-induced acute kidney injury (CI-AKI) is a decline in renal function due to iodinated contrast administration and occurs more frequently in individuals with increasingly common risk factors, such as diabetes mellitus (DM). Physical training (PT) can have renoprotective effects on CI-AKI in diabetic nephropathy. The aim of this study was to evaluate the injury in kidneys of diabetic rats submitted to treatment with IC, evaluating the impact of PT on hemodynamics and renal function in addition to oxidative profile in diabetic rats submitted to IC-AKI.

**Materials and Methods:**

Adult male Wistar rats are randomized into four groups: citrate (*n* = 7): control group, citrate buffer (streptozotocin-STZ vehicle), intravenous tail (iv), single dose; DM (*n* = 7): STZ, 60 mg/kg, iv, single dose; DM+IC (*n* = 7): DM rats treated with IC (sodium meglumine ioxithalamate, 6 mL/kg, intraperitoneal (ip), single dose); DM+IC+PT (*n* = 7): DM rats treated with IC as mentioned and submitted to physical training. Renal function parameters (inulin clearance, neutrophil gelatinase-associated lipocalin (NGAL), serum creatinine, and urinary albumin), hemodynamics (renal blood flow and renal vascular resistance), and oxidative profile (urinary peroxides, urinary TBARS, urinary nitric oxide, and renal tissue thiols) were evaluated.

**Results:**

It was possible to observe a decrease in inulin clearance, renal blood flow, and thiols in renal tissue accompanied by an increase in urinary flow, serum creatinine, urinary albumin, renal vascular resistance, urinary peroxides, urinary nitrate, and TBARS in the DM group compared to the citrate group. The DM+IC group showed a reduction in inulin clearance, and the renal dysfunction was also seen by the increased NGAL. Renal hemodynamics and oxidative profile compared were also worsened in the DM group. PT improved renal function by increasing renal blood flow and thiol levels in renal tissue and reduced renal vascular resistance, metabolites of reactive oxygen, nitrogen species, and lipid peroxidation in the DM+IC+PT group compared to DM+IC.

**Conclusions:**

Our results confirmed that DM induction increases renal vulnerability to the toxicity of IC and an association between DM with IC predisposes to severe AKI with reduced renal function alongside with renal hemodynamic alterations and oxidative mechanism of injury. The PT showed a renoprotective effect in DM animals subjected to damage with IC by modulating renal hemodynamics and oxidative profile, confirming a potential to modify the risk of CI-AKI when diabetes mellitus is present.

## 1. Introduction

Iodinated contrast (IC), used to improve the visibility of organs and structures in diagnostic exams, is a leading cause of acute kidney injury (AKI) [1]. Contrast-induced acute kidney injury (CI-AKI) is the third leading cause of AKI in hospitalized patients and is clinically defined by an increase in serum creatinine ≥ 0.5 mg/dL or 25% increase in serum creatinine from the baseline value 48 h past the contrast media administration [2, 3].

CI-AKI increases morbidity and mortality risk and occurs more frequently in the presence of risk factors, such as preexisting renal impairment or diabetes mellitus (DM), with prevalence estimated at approximately 30% [4, 5].

Pathophysiology mechanisms for CI-AKI are complex and remain unclear, but it is known that it involves cell damage caused by the direct cytotoxic effect of IC to renal tubular epithelial cells, renal vasoconstriction resulting in medullary hypoxia, and the formation of reactive oxygen species (ROS) [1, 6].

CI-AKI has been growing in the recent years and will probably increase its prevalence in the future with the more frequent contrast-mediated diagnostic need in old patients and with increasingly common comorbid conditions such as DM [3, 5]. There is no specific treatment for CI-AKI; therefore, strategies to recover kidney function and reduce the CI-AKI risk in the presence of DM are urgently needed [3, 6].

Physical training (PT), a nonpharmacological therapy recommended to control DM, can be promising to attenuate the complications of aggravated CI-AKI in DM because it induces beneficial adaptive responses and influences physiological biomarkers, such as oxidative stress biomarkers, nitrosative stress, and hemodynamics, which implicate in the development and progression CI-AKI [7, 8].

Recently, researchers have been investigating the role of PT on AKI and the favourable effects observed reinforce its role in preventing AKI [9–11]. Although many studies have shown the beneficial influence of PT in several diseases, the mechanisms involved are not clear and little is known about this process in the CI-AKI, which reinforces the need for further study in this field.

In this study, we have investigated the injury in kidneys of diabetic rats submitted to treatment with IC, evaluating the PT impact on hemodynamics and renal function in addition to oxidative profile in diabetic rats submitted CI-AKI.

## 2. Materials and Methods

### 2.1. Animals

Adult male Wistar rats (weighing 250-290 g) were used. The animals were obtained from the Institute of Biomedical Sciences at the University of Sao Paulo and were housed at the Experimental Laboratory of Animal Models (LEMA) at the School of Nursing at the University of Sao Paulo, in a room with controlled temperature (25°C/77°F) and alternating light/dark cycles. The rats had free access to water and rat chow. The study was carried out in accordance with international standards for the manipulation and care of laboratory animals. The protocol was approved by the Ethical Committee of Experimental Animals, University of Sao Paulo (CEEA–protocol n. no. 1277/2019).

### 2.2. Streptozotocin-Induced Diabetes Mellitus Model

The animals received streptozotocin (STZ; 60 mg/kg), diluted in 0.5 mL of citrate buffer (0.1 mol/L; pH 4.5) in the first day of the protocol. The injection was administrated in the caudal vein for DM induction. The control animals received only 0.5 mL of citrate buffer. Blood glucose levels were measured 48 h after the injection to confirm hyperglycemia (Accu-Chek, Roche; measurement range: 10–600 mg/dL). Animals considered hyperglycemic and included in the study were those that consistently showed a blood glucose level higher than 250 mg/dL [12]. DM induced in this protocol is considered type I DM.

### 2.3. Iodinated Contrast Administration

The animals received 6 mL/kg of IC (meglumine ioxithalamate and sodium) i.p., single dose on the 26th day of experimental protocols [13].

### 2.4. Physical Training

This protocol includes an acclimation period of 1 week to the aquatic environment after DM induction and before PT. PT consisted of daily swimming, five days/week, 60 minutes/day, with an additional load corresponding to 5% of the animal's body weight placed in the tail. This protocol is considered moderate-intensity aerobic PT [14–16].

### 2.5. Experimental Groups

- Citrate (*n* = 7): control group of chronic diabetes mellitus model, rats that received 0.5 mL of citrate buffer (STZ vehicle), intravenous (iv) tail, single dose on 1st day.
- Diabetes mellitus (DM, *n* = 7): rats receiving 60 mg/kg of STZ diluted in 0.5 mL citrate buffer, iv tail, single dose.
- Diabetes mellitus+iodinated contrast (DM+IC, *n* = 7): rats receiving 60 mg/kg STZ diluted in 0.5 mL citrate buffer iv tail and received 6 mL/kg of IC intraperitoneal (ip), single dose on 26th day.
- Diabetes mellitus+iodinated contrast+physical training (DM+IC+PT, *n* = 7): rats receiving 60 mg/kg STZ diluted in 0.5 mL citrate buffer iv and daily swimming, five days/week, 60 minutes/day from the 6th day and received 6 mL/kg of IC ip, single dose on 26th day.

### 2.6. Procedures and Timing of Experimental Protocols

All protocols of experimental groups lasted 28 days. PT was performed until the 26th day for the animals in the trained group, while the other nonexercised animals were kept in collective cages during this period. On the 27th protocol day, the animals were allocated in individual metabolic cages for 24 hours, for collection of urine samples and determination of urinary flow.

The rats were removed from the individual metabolic cages on the 28th day of the protocol and anesthetized with 10 mg/kg xylazine and 90 mg/kg ketamine ip and tracheostomized to maintain spontaneous breathing during experiments for renal function and hemodynamic measurements, performed through a catheter inserted into the left carotid artery and in the right jugular vein (polyethylene tube PE-60). An abdominal incision was made, and the urinary bladder was cannulated (polyethylene tube PE-240).

After the surgical procedure and the obtaining of the parameters for renal hemodynamics, a blood sample was collected through a puncture of the distal abdominal aorta. Finally, animals were submitted to terminal blood collection and euthanasia according to guidelines for animal experimentation. The right kidney was removed and immediately cooled and stored at -70°C for thiol assay.

### 2.7. Renal Function Measurement

- Inulin clearance (mL/min): renal function was evaluated based on inulin clearance. Inulin was injected in the right jugular vein, with a loading dose of 100 mg/kg, followed by a continuous infusion of 0.04 mL/min. After a 30 min stabilization period, three urine samples were collected through the bladder catheter and two blood samples were then collected through the carotid catheter. The serum and urine inulin were measured using the anthrone method [17, 18]
- Urinary neutrophil gelatinase-associated lipocalin (NGAL, pg/mL): urinary NGAL was determined using the Rat NGAL ELISA Kit, BioVendor, research and diagnostic products
- Serum and urinary creatinine (Cr, mg/dL): serum and urinary creatinine were measured using the Jaffe method. The results were expressed in mg/dL; oxidative parameters were corrected using urinary creatinine [19]
- Urinary albumin (mg/24 h): albuminuria was determined using the Rat Albumin ELISA Kit, Bethyl Laboratories

### 2.8. Hemodynamic Measurements

- Heart rate (beats per minute (bpm)): heart rate was measured through the catheter inserted into the carotid artery and was assessed using Biopac Systems MP150 (Santa Barbara, CA)
- Mean arterial blood pressure (mmHg): mean arterial blood pressure was measured through the catheter inserted into the carotid artery and was assessed using Biopac Systems MP150 (Santa Barbara, CA)
- Renal blood flow (mL/min): the renal artery was isolated after exposing the left renal pedicle, and a suitable probe was placed around for renal blood flow measurement, which was performed by a perivascular ultrasonic flowmeter (T402, Transonic Systems Inc., Bethesda, MD)
- Renal vascular resistance (mmHg/mL/min): mean arterial blood pressure and renal blood flow were assessed, and renal vascular resistance was calculated with the usual formula: renalvascularresistance = meanarterialbloodpressure/renalbloodflow [17]

### 2.9. Oxidative Profile: Oxidative and Nitrosative Metabolites, Lipid Peroxidation, and Thiol Antioxidant Assay

- Urinary peroxides (nmol/g urinary Cr): the peroxides, ROS metabolites, were determined in the urinary samples by the method of ferrous oxidation of xylenol orange version 2 [20]
- Urinary nitrate (*μ*M/g urinary Cr): the urinary nitrate excretion, RNS metabolites, was measured using the Griess reaction method [21]
- Thiobarbituric acid reactive substances (TBARS, nmol/g urinary Cr): urinary TBARS were evaluated as an indirect biomarker of lipid peroxidation. Urine samples were added to a mixture of 17.5% trichloroacetic acid (TCA) and 0.6% thiobarbituric acid. This mixture was heated to 95°C in a water bath for 20 min. The solution was removed from the water bath and cooled in ice, followed by the addition of 70% TCA. The solution was then incubated for 20 min, and absorbance was read at 534 nm [22]
- Thiols in renal tissue (nmol/mg total protein): the soluble nonprotein thiols in renal tissue were measured by the thiol antioxidant assay using the Ellman method. The amount of soluble thiols was corrected to the total protein amount analyzed using the Bradford method [23, 24]

### 2.10. Statistical Analysis

Results were expressed as mean ± standarddeviation. Variance was analyzed using the one-way ANOVA test, followed by Newman-Keuls (GraphPad Prism version-7 for Windows®): ^a^*p* < 0.001 vs. citrate; ^b^*p* < 0.001 vs. DM; ^c^*p* < 0.001 vs DM+IC.

## 3. Results and Discussion

### 3.1. Effects of Physical Training on Renal Function

To evaluate the effectiveness of PT on CI-AKI on diabetic nephropathy, glomerular filtration rate was assessed by inulin clearance studies and changes on urinary flow, urinary NGAL, serum creatinine, and urinary albumin.

The DM induced in this study resulted in worsening renal function as demonstrated by decreased inulin clearance and increased urinary flow, serum creatinine, and albuminuria (Table 1).

The administration of IC to diabetic rats caused an additional deleterious effect on renal function as demonstrated by increased NGAL in the DM+IC group (Table 1). Among markers of renal function, inulin clearance is the most reliable to estimate glomerular filtration rate while NGAL has been described as a sensitive and specific marker of AKI [25, 26]. Therefore, the increase of NGAL might be associated with the onset of CI-AKI in the diabetic animals.

Initially, IC acts as an osmotic diuretic, freely filtered by the glomeruli and poorly absorbed by the renal tubule [27]. However, the IC cytotoxic effect to renal cells of tubular epithelial increases the viscosity of tubular fluid, compromising its flow, which leads to further renal retention and greater cytotoxic exposure [28]. IC results in a long-term effect on perfusion and oxygenation throughout the kidney, and according to clinical observations, it has been verified that the serum Cr level in patients with CI-AKI oftentimes reaches the peak within 2–5 days after IC administration [28].

Albuminuria, which occurs due to microvascular dysfunction in DM, is a biomarker for chronic kidney disease, and it is associated with a greater risk of AKI even in patients with a preserved renal function after contrasted exam [29, 30]. Lifestyle modification program, including physical exercise, demonstrated reduction of albuminuria and maintenance of glomerular filtration rate in diabetic patients [31].

In this study, IC induced a reduction in glomerular filtration rate in the animals with diabetic nephropathy as demonstrated by decreased inulin clearance. PT prevented the sharp reduction in the inulin clearance caused by IC in the diabetic animals. In addition, PT prevented the worsening of renal function as observed by lower NGAL, serum creatinine, and urinary albumin in DM+IC+PT compared with DM+IC (Table 1). This is probably due to adaptive mechanisms of renal function and to the adaptation to physical stress acquired gained with training.

Our results suggest that PT is a good, nonpharmacological strategy to preserve renal function and reduce the CI-AKI risk in the presence of diabetic nephropathy. Although studies have shown the beneficial influence of physical exercise in several diseases, the mechanisms involved are not clear. To clarify how PT could contribute to kidney protection, renal hemodynamics and renal oxidative profile were further analyzed.

### 3.2. Effects on Renal Hemodynamics

A statistically significant difference was not detected in the mean arterial pressure between groups. There was a significant decrease in heart rate in the DM+IC+PT group after physical training compared to the DM+IC group without training (Table 2).

DM showed a deleterious effect on renal hemodynamics with a decrease in renal blood flow and an increase in renal vascular resistance (Table 2). The use of IC worsened the effects of DM on the renal blood flow and vascular resistance (Table 2). However, PT prevented an excessive increase in vascular resistance and a decrease in renal blood flow caused by IC (Table 2).

The association of direct medullary vasoconstriction and high viscosity of IC compromises cell oxygen delivery and reduces intrarenal blood flow. Animal experiments demonstrated a reduction in renal blood flow induced by IC partly assigned by the downregulation of endogenous renal NO synthesis [28].

Renal hemodynamic dysfunctions are observed in AKI and are involved in the establishment and evolution of renal injury. Therefore, stabilization of hemodynamic parameters is crucial to the management of CI-AKI [3]. In the present study, PT attenuated renal hemodynamic changes in diabetic nephropathy animals submitted to IC damage, probably by decreasing renal vascular resistance, which resulted in improved renal blood flow.

### 3.3. Effects of Oxidative Profile

To determine whether the antioxidant effect of PT is involved in renal protection in the current experimental model, urinary peroxides, urinary nitrate, serum TBARS (a byproduct of lipid peroxidation), and thiol levels in renal tissue (indicative of reduced glutathione, a major endogenous antioxidant) were assessed.

It was observed a significant increase in urinary peroxides and nitrate as well as in TBARS levels in the diabetic group, whereas thiols were significantly reduced compared to the citrate group (Table 3). Additionally, IC administration worsened the oxidative profile in diabetic rats (Table 3).

The IC vasoconstriction results in hypoperfusion and decreased oxygen level in the inner stripes of the outer medulla, causing severe local hypoxia, contributing to oxidative and nitrosative stress, which is known to be mediated by ROS and reactive nitrogen species (RNS) [32]. Data demonstrated that oxidative stress, nitrosative stress, and lipid peroxidation increase in the kidney of diabetic animals were worsened after IC treatment. This could be due to the decrease in antioxidant enzymes, such as glutathione, a thiol enzyme.

The decrease in thiols in the kidney and the increase in lipid peroxidation in DM+IC were significantly improved after exercise. PT showed an improvement in oxidative profile with a significant reduction in oxidative and nitrosative metabolites (peroxides, TBARS, and urinary nitrate) and a significant increase in thiol antioxidant reserve in DM+IC+PT when compared to DM+IC (Table 3).

PT has been used to prevent or treat chronic degenerative conditions once it promotes cellular responses signaled by the physiological stress of exercise [3, 33]. Studies that analyzed the effects of moderate physical exercise with vitamin supplementations on oxidative stress in diabetic rats demonstrated that this association can strengthen the antioxidant defense system through the reduction of ROS and blood glucose levels, preventing the development of diabetic nephropathy [33].

The data collected in this study highlight PT potent antioxidant action in IC-AKI, demonstrating that PT decreases the vulnerability of the diabetic kidney to acute damage due to IC treatment, contributing to an improvement in the renal oxidative profile, with reduced oxidative and nitrosative metabolites and elevated the thiol levels.

## 4. Conclusions

Our results confirmed that DM induction increases renal vulnerability to the toxicity of IC. The association of DM and IC predisposes to severe AKI with reduced renal function in rats by changing renal hemodynamics and oxidative mechanism of injury. This study highlights the exercise-induced antioxidant effect on IC nephrotoxicity in diabetes mellitus in experimental settings, by modulating renal hemodynamics.

## Figures and Tables

**Table 1:** Renal function.

Groups	*n*	Urinary flow (mL/min)	Inulin clearance (mL/min)	Urinary NGAL (pg/mL)	Serum creatinine (mg/dL)	Urinary albumin (mg/24 h)
Citrate	7	0.012 ± 0.002	0.83 ± 0.09	44.06 ± 10.15	0.30 ± 0.06	3.76 ± 0.91
DM	7	0.055 ± 0.011^a^	0.49 ± 0.10^a^	73.11 ± 29.24	0.92 ± 0.06^a^	39.42 ± 8.89^a^
DM+IC	7	0.074 ± 0.006^ab^	0.16 ± 0.05^ab^	163.64 ± 35.08^ab^	1.35 ± 0.15^ab^	71.37 ± 8.88^ab^
DM+IC+PT	7	0.093 ± 0.019^abc^	0.59 ± 0.05^ac^	84.39 ± 7.36^ac^	1.05 ± 0.07^ac^	40.95 ± 9.44^ac^

^a^ *p* < 0.001 vs. citrate; ^b^*p* < 0.001 vs. DM; ^c^*p* < 0.001 vs. DM+IC.

**Table 2:** Renal hemodynamics.

Groups	*n*	Heart rate (bpm)	Mean arterial blood pressure (mmHg)	Renal blood flow (mL/min)	Renal vascular resistance (mmHg/mL/min)
Citrate	7	509 ± 39	96 ± 6	7.47 ± 1.49	12.56 ± 2.19
DM	7	532 ± 48	102 ± 8	4.03 ± 0.27^a^	25.72 ± 1.82^a^
DM+IC	7	539 ± 44	102 ± 16	1.70 ± 0.19^ab^	54.35 ± 6.34^ab^
DM+IC+PT	7	464 ± 27^c^	95 ± 5	4.20 ± 0.44^ac^	24.02 ± 5.04^ac^

^a^ *p* < 0.001 vs. citrate; ^b^*p* < 0.001 vs. DM; ^c^*p* < 0.001 vs. DM+IC.

**Table 3:** Urinary peroxides, urinary nitrate, TBARS, and thiols.

Groups	*n*	Urinary peroxides (nmol/g urinary Cr)	Urinary nitrate (*μ*M/g urinary Cr)	TBARS (nmol/g urinary Cr)	Thiols (nmol/mg total protein)
Citrate	7	1.33 ± 0.82	23.71 ± 4.96	0.22 ± 0.15	24.45 ± 5.39
DM	7	11.64 ± 4.00^a^	56.82 ± 12.73^a^	12.91 ± 3.02^a^	13.65 ± 1.73^a^
DM+IC	7	19.96 ± 6.98^ab^	75.32 ± 9.65^ab^	20.49 ± 5.29^ab^	8.56 ± 1.17^ab^
DM+IC+PT	7	10.84 ± 2.75^ac^	51.75 ± 7.66^ac^	14.10 ± 2.31^ac^	14.45 ± 1.88^ac^

^a^ *p* < 0.001 vs. citrate; ^b^*p* < 0.001 vs. DM; ^c^*p* < 0.001 vs. DM+IC.

## Data Availability

The research article data used to support the findings of this study are available from the corresponding author upon request.
